# pH-Controlled Yeast Protein Precipitation from *Saccharomyces cerevisiae*: Acid-Induced Denaturation for Improved Emulsion Stability

**DOI:** 10.3390/foods14152643

**Published:** 2025-07-28

**Authors:** Laura Riedel, Nico Leister, Ulrike S. van der Schaaf

**Affiliations:** Institute of Process Engineering in Life Sciences—Food Process Engineering, Karlsruhe Institute of Technology, 76131 Karlsruhe, Germany; laura.riedel@kit.edu (L.R.); nico.leister@kit.edu (N.L.)

**Keywords:** single cell proteins, emulsifier, protein extraction, protein precipitation, acid denaturation, emulsions, functional properties, alternative proteins, yeast

## Abstract

In the search for alternative protein sources, single cell proteins have gained increasing attention in recent years. Among them, proteins derived from yeast represent a promising but still underexplored option. To enable their application in food product design, their techno-functional properties must be understood. In order to investigate the impact of precipitation pH on their emulsion-stabilizing properties, yeast proteins from *Saccharomyces cerevisiae* were isolated via precipitation at different pH (pH 3.5 to 5) after cell disruption in the high-pressure homogenizer. Emulsions containing 5 wt% oil and ~1 wt% protein were analyzed for stability based on their droplet size distribution. Proteins precipitated at pH 3.5 stabilized the smallest oil droplets and prevented partitioning of the emulsion, outperforming proteins precipitated at higher pH values. It is hypothesized that precipitation under acidic conditions induces protein denaturation and thereby exposes hydrophobic regions that enhance adsorption at the oil–water interface and the stabilization of the dispersed oil phase. To investigate the stabilization mechanism, the molecular weight of the proteins was determined using SDS-PAGE, their solubility using Bradford assay, and their aggregation behavior using static laser scattering. Proteins precipitated at pH 3.5 possessed larger molecular weights, lower solubility, and a strong tendency to aggregate. Overall, the findings highlight the potential of yeast-derived proteins as bio-surfactants and suggest that pH-controlled precipitation can tailor their functionality in food formulations.

## 1. Introduction

The global population is increasing, leading to a rising demand for food and food components such as proteins [1,2]. In order to adapt the production of proteins to current challenges such as the climate crisis, it should be as sustainable and environmentally friendly as possible. In addition to plant sources, single-cells like yeasts are a good option. The latter offer the advantages that they can be cultivated in a decentralized manner and independently of weather conditions, which is particularly advantageous in the context of climate change [3]. Additionally, yeast can be produced using by-products and side-streams of food production, such as molasses as an energy source. Moreover, they are already accepted by consumers due to their long use in brewing and baking [4,5,6,7,8].

According to literature values, the dry matter of yeast cells consists of approximately 40–60 wt% proteins [9,10,11]. The proteins are present both inside the cell, dissolved in the cytoplasm, and outside, bound to the cell wall in the form of mannoproteins. To the best of our knowledge, there is no precise information in the literature on the proportions in which the proteins are distributed between these two groups.

It is generally known that proteins have surface-active properties because they possess both hydrophobic and hydrophilic molecular regions. Due to this structure, they are able to stabilize (oil-in-water) emulsions, hence they are often used in food as emulsifiers or emulsion-stabilizing ingredients [12,13]. Mannoproteins, obtained from the wall of yeast cells, have been described several times as effective emulsion stabilizers [14,15,16,17]. In contrast, intracellular proteins have not yet been studied as extensively. However, their emulsion stabilizing properties have been previously demonstrated [18,19]. The intracellular proteins can be made accessible by cell disruption via high-pressure homogenization (HPH) [18,20,21,22,23,24,25]. In our recent study [26], we showed that formulation parameters known to influence the emulsifying performance of proteins, in general, also affect the performance of yeast proteins. The protein-rich supernatant, obtained after cell disruption and subsequent separation of the cell wall fragments, was used to stabilize oil-in-water emulsions at different pH. At pH 3 and 7, stable emulsions with an oil content of up to 50 wt% could be produced. However, at pH 5, the emulsion partitioned immediately after production. The creaming of the drops at pH 5 cannot be attributed solely to the lower electrostatic stabilization near the isoelectric point (IEP) (approx. pH 4), since the amounts of the zeta potentials at pH 3 and 5 are approximately the same [26]. Especially at pH 3, emulsions were stable over a longer period of time, i.e., several weeks, despite the low zeta potential of the proteins. It was hypothesized that, in contrast to pH 7, the acidic conditions at pH 3 led to a denaturation of the proteins and thus to the exposure of hydrophobic groups from the interior of the protein. The larger number of available hydrophobic groups after acid denaturation of globular proteins was previously described to allow for a better and faster stabilization of oil droplets [12,27,28].

However, a pH value of 3, is not suitable for various food applications, e.g., milk substitutes, that typically have a neutral pH value [29]. Therefore, the question arises if the positive effect of acid denaturation during protein precipitation can be used to enhance yeast protein functionality at pH 7.

Currently, protein precipitation from yeast is performed at the IEP of the proteins, at pH values between 4.0 and 4.5, to obtain intracellular proteins [18,30,31]. Only Haehn differentiated between two fractions of yeast albumin with an IEP of 4.59 and 3.6, respectively [32].

Unfortunately, the properties of the proteins are only comparable to a limited extent, since they are not only precipitated at different pH values, but also the yeast strain, digestion method, etc., vary throughout the publications. In addition, “yeast proteins from inside the cell” are not just one protein, but always a mixture, the composition of which can also vary due to the fermentation and storage conditions. Each protein fraction of this mixture can have a different IEP and different functional properties.

This raises the question of how the pH value of precipitation affects the yield and techno-functional properties of the intracellular protein mixture; in particular, its ability to stabilize emulsions, when other parameters such as yeast strain, cultivation conditions, and processing steps, e.g., cell disruption and drying, are held constant.

## 2. Materials and Methods

Unless otherwise indicated, demineralized water was used for the experiments.

### 2.1. Yeast Cells (Saccharomyces cerevisiae)

Fresh baker’s yeast was bought in cubes from a local supermarket. The manufacturer was Lesaffre Deutschland GmbH (Kehl, Germany), and the yeast cubes were sold under the brand name “Omas Urhefe”. The manufacturer was contacted but refrained from disclosing the precise yeast strain. The cubes were stored at −23 °C until needed. For further use, the required cubes were thawed in the refrigerator (6 °C) overnight.

### 2.2. Cell Disruption and Protein Precipitation

For cell disruption, 20 wt% yeast cubes were dispersed in demineralized water, and the pH value was adjusted to pH 7 using NaOH obtained from Carl Roth GmbH + Co. KG (Karlsruhe, Germany) to keep the proteins in solution after cell rupture. The dispersion was cooled to 13 °C in an ice bath before high-pressure homogenization (HPH) to avoid excessive heating during processing. The dispersion was subjected to a single pass through a high-pressure homogenizer at 1200 bar (Microfluidizer M-110EH with a Y-reaction chamber, Microfluidics Corporation, Newton, MA, USA). Following cell disruption, the suspension was brought to room temperature and centrifuged at 4250 rpm for 15 min at 20 °C in an Eppendorf 5920 R centrifuge (Eppendorf SE, Hamburg, Germany). The resulting supernatant, containing the soluble proteins, was decanted, and the pH value was adjusted to 3.5, 4.0, 4.5, or 5.0 with HCl obtained from Carl Roth GmbH + Co. KG (Karlsruhe, Germany). After 2 h, the precipitated protein was separated from the soluble components by centrifugation (see above). All protein precipitates were uniformly resuspended in water at pH 7 and to prevent differences that could potentially be caused by cell-derived proteases during storage, the samples were frozen and lyophilized in a freeze-drier from Becker Technology GmbH (Eschborn, Germany).

### 2.3. Protein Yield

The weight of the lyophilized proteins was determined gravimetrically after drying. The yield was calculated using Equation (1) and is given in percent:

protein yield = m _lyophilized protein powder_/m _yeast biomass dispersed for cell disruption_ × 100 (1)

The proteins were ground for further use in the household mill KSW 3307 from Clatronic (Kempen, Germany), and the powders were stored in opaque containers in the refrigerator at 6 °C.

### 2.4. Protein Solubility

First, 0.05 wt% of the lyophilized yeast proteins were redispersed in demineralized water at pH 7 on the shaking table for 2 h. Then the samples were centrifuged (20 °C, 4250 rpm, Eppendorf 5920 R, Hamburg, Germany).

The amount of dissolved protein in the supernatant was determined using the Bradford assay [33]. Therefore, 1 mL of the Quick Start Bradford 1× Dye Reagent from Bio-Rad Laboratories Inc. (Hercules, CA, USA) was mixed with 20 µL of the supernatant. After 15 min, the extinction was determined photometrically at 595 nm in the Evolution 201 UV-vis-spectrophotometer by Thermo Scientific (Waltham, MA, USA). The calibration was conducted with bovine serum albumin (BSA) obtained from Carl Roth GmbH + Co. KG (Karlsruhe, Germany). Protein solubility was calculated by dividing the determined amount of dissolved protein in the supernatant by the weighed amount of lyophilized protein.

To investigate the influence of an additional HPH-treatment, the lyophilized proteins were first dispersed at pH 7 for 2 h (see above) and then pumped through the high-pressure homogenizer Microfluidizer M-110EH with Y-reaction chamber at 400 bar (Microfluidics Corporation, Newton, MA, USA) before centrifugation and subsequent Bradford assay.

### 2.5. Zeta Potential

The zeta potential of the proteins in the supernatant after cell disruption was measured at pH values between 3.0 and 6.0 in steps of 0.5.

For that, the supernatant was diluted 1:5 and the pH value was adjusted with HCl obtained from Carl Roth GmbH + Co. KG (Karlsruhe, Germany). The electrophoretic mobility was measured with the Nanopartica SZ-100Z by Horiba Scientific (Kyoto, Japan) at 25 °C. The in-built software used the Smoluchowski model for calculating the zeta potential from the measured values of the electrophoretic mobility. The electrophoretic mobility was measured tenfold each time. The IEP was determined by reading out the intersection of the interpolated zeta potential curve with the *x*-axis.

### 2.6. SDS-PAGE

The running buffer used to execute the SDS-PAGE, consisted of the following components: 15.15 g Tris (tris(hydroxymethyl)aminomethane), 72.00 g glycine, and 5.00 g SDS (sodium dodecyl sulfate) (all obtained from Carl Roth GmbH + Co. KG, Karlsruhe, Germany), dissolved in 500 mL bi-distilled water.

In addition, Mini-PROTEAN TGXTM gels, Precision Plus Protein Standards All Blue and 4× Laemmli Sample Buffer from Bio-Rad Laboratories Inc. (Hercules, CA, USA) were used.

A total of 1 wt% of the precipitated and dried proteins were mixed with bi-distilled water for 2 h on the shaking table and then diluted with the sample buffer in a ratio of 1:8.5. After 5 min in the water bath at 95 °C, the protein solutions were loaded onto the gel and separated in the Mini-PROTEAN Tetra Cell from Bio-Rad Laboratories Inc. (Hercules, CA, USA) at 200 V for about 30 min. After washing with bi-distilled water three times, the gels were placed in the Coomassie Brilliant Blue R-250 Staining Solution from Bio-Rad Laboratories Inc. (Hercules, CA, USA) for 1 h and afterwards again washed with water three times. To remove all excess color from the gel, they were additionally treated with Coomassie Brilliant Blue R-250 Destaining Solution and then photographed.

This determination was conducted only once, as is usual for an SDS-PAGE.

### 2.7. Protein Particle Size Distribution

A total of 1 wt% lyophilized yeast protein was dispersed in water at pH 7 on the shaking table for 2 h. For the samples prepared to analyze the effect of HPH treatment, a subsequent pass through the HPH at 400 bar was performed. Afterwards, all samples were further analyzed. The size of the protein particles was determined using static laser light scattering (SLS) in the standing measuring cell of the particle analyzer (LA-950 from Horiba, Microtrac Retsch GmbH, Haan, Germany).

For the protein, a refractive index of 1.450 [18] and for water of 1.333 was set. The particle sizes are presented as the volumetric cumulative size distribution Q_3_ or its 90th percentile (d_90,3_).

### 2.8. Emulsion Preparation

All emulsions contained 5 wt% rapeseed oil obtained from B. Schell (Lichtenau (Baden), Germany) as the disperse phase and 0.95 wt% of lyophilized protein as emulsifier.

For the continuous phase of the emulsions, 1 wt% protein was dissolved in water at pH 7 for 2 h or, if necessary, additionally homogenized (see above). A total of 142.5 g of the continuous phase was taken, and 7.2 g of the rapeseed oil was added to prepare 150 g of an emulsion premix. To do so, the oil was added while stirring with an Ultra Turrax T 25 digital by IKA (Staufen im Breisgau, Germany) at 12,000 rpm (7.85 m/s) for 30 s. Following this, this premix was pumped through the high-pressure homogenizer and emulsified at 400 bar for one pass (Microfluidizer M-110EH with Y-reaction chamber (Microfluidics Corporation, Newton, MA, USA)).

### 2.9. Droplet Size Distribution

Droplet size distributions (DSDs) were determined using SLS as described for the measurement of the protein particle size distributions. For rapeseed oil, a refractive index of 1.470 was used.

DSDs were determined again after storing the emulsions at 6 °C for 14 days in order to evaluate the long-term stability of the emulsions.

### 2.10. Microscopic Images

Microscope images of the emulsions were taken with the Axiolab re 450905 microscope and the Axiocam 105 color (Carl Zeiss AG, Oberkochen, Germany). The respective emulsion was sampled from the center of the sample vial using a plastic pipette. A single emulsion droplet was then placed in the middle of a slide and carefully covered with a coverslip.

### 2.11. Statistical Analysis

Unless otherwise stated, measurements were performed in triplicate. Statistical analyses were performed using Origin 2020 (OriginLab Corp., Northampton, MA, USA). The results in this work are presented as means ± standard deviation. For the ANOVA, the Scheffe test was used to compare the means, and the Brown–Forsythe test was used to check the homogeneity of the variances.

## 3. Results

The following section shows and discusses the results of this study. First, the supernatant after cell disruption and the effects of the different pH values during precipitation on the yield of the proteins and their properties in an aqueous environment are discussed. Then, the results of the emulsification experiments are presented, and various hypotheses are discussed that could explain the differences in the emulsion-stabilizing properties after precipitating the proteins at different pH values. Furthermore, the influence of an additional homogenization treatment on the proteins and their emulsion stabilization properties is described and discussed.

### 3.1. Examination of the Protein-Containing Supernatant and Influence of Precipitation pH on Protein Yield

Before investigating the effect of precipitation pH, the protein content and zeta potential of the supernatant directly after cell disruption were measured. The protein content in the supernatant after cell disruption was measured to be 12.1 ± 0.3 mg BSA-equivalent/mL. By measuring the zeta potential (see Figure S1 in the Supplementary Materials), an IEP of about 4.5 could be determined for the proteins in the supernatant. This value corresponds to the values described in the literature for yeast proteins (s. Introduction). Therefore, for the following protein precipitation, pH values were set around the IEP, namely from 3.5 to 5 in 0.5 steps.

**Figure 1:**
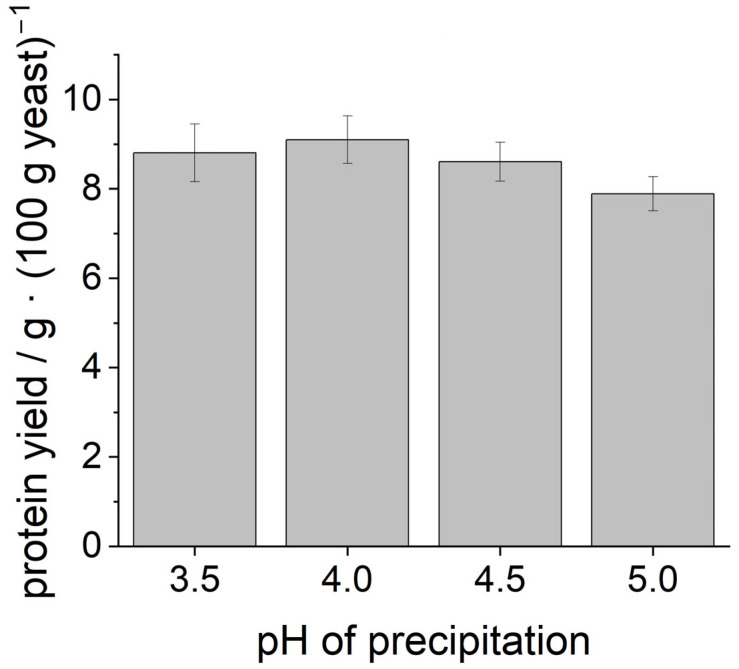
Protein yield from 100 g of fresh yeast after cell disruption, precipitation at different pH values, and lyophilization.

Figure 1 shows the yield of dry protein powder that could be obtained. For all precipitation pH values, a protein yield of about 8–9 g from 100 g fresh yeast could be determined. ANOVA indicated that there was no statistically significant difference in the yields.

In total, yeast cells contain 40–60 g protein in 100 g dry matter [9,10,11]. Since fresh yeast has a dry matter content of 30% [16], a total protein content of 12–18 g protein can be calculated for 100 g of fresh yeast, as used in this study.

The yields achieved in this study are below those described in the literature. This can be explained by the fact that not all yeast proteins are present in the dissolved form inside the cell, but are also bound to the cell wall in the form of mannoproteins. These mannoproteins are separated along with the other cell wall components after cell disruption and are not contained in the precipitated protein. To the best of our knowledge, there is no information in the literature on how the proteins within the cell are distributed between the cell wall and the cell interior. Furthermore, it should be noted that maximizing protein yield was not the focus of this study.

From the comparable protein yield at different precipitation pH, one might assume that also the functional properties of the samples will be comparable. However, intracellular proteins do not consist of only a single protein fraction. They are rather a mixture of different proteins with slightly varying IEP [32]. Therefore, it is theoretically possible that a slight variation in the precipitation pH causes one protein fraction to precipitate more while another fraction will precipitate less, ultimately resulting in the same overall yield. This might result in varying functionalities of the lyophilized protein samples depending on the amount of the individual protein fractions. Therefore, as described below, we investigated how the different precipitated proteins differ in their properties.

### 3.2. Influence of the Precipitation pH on the Precipitated Protein Molecules

**Figure 2:**
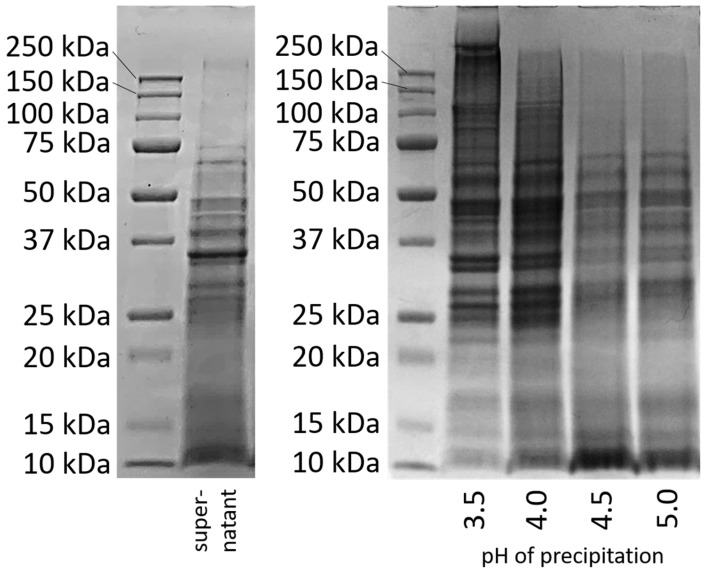
SDS-PAGE of the supernatant (**left**) [26] and after protein precipitation at pH 3.5–5.0 and redissolution in water at pH 7 for 2 h (**right**).

To obtain a better insight into the types of precipitated proteins, an SDS-PAGE was conducted with the proteins precipitated at different pH values. The results are compared in Figure 2 with the band pattern of the protein solution, which was prepared directly after cell disruption without further pH treatment [26].

The band pattern of the supernatant after cell disruption (Figure 2 left) shows an intense coloration at 10 kDa and above up to a molecular weight of about 75 kDa, with the most obvious band at about 35 kDa. Above a molecular weight of 75 kDa, only very weak bands can be seen. Figure 2 right shows the band patterns for the proteins that had precipitated from the supernatant at pH 3.5, 4.0, 4.5, and 5.0, had then been freeze-dried and again dissolved/dispersed in water at pH 7 for 2 h.

At a precipitation pH of 3.5, an intense coloration at high molecular weights (>75 kDa) and very high molecular weights (>150 kDa) can be observed, which was not visible in the supernatant. Also, after precipitation at pH 4.0, bands above 75 kDa were visible that had not yet been visible in the supernatant. However, the intensity of the bands in the very high molecular weight region was lower. The bands at higher molecular weights (>75 kDa) that were previously not visible in the supernatant indicate covalent linkages due to disulfide bonding.

Disulfide bonding of proteins at low pH can be caused by acid denaturation [28]. Acid denaturation can lead to an unfolding of protein molecules and thus to the exposure of the hydrophobic and reactive sulfide groups located inside. The exposed sulfide groups could then react with one another, leading to covalent bonds. This phenomenon was previously described for intracellular yeast proteins by our group [26].

In contrast, samples precipitated at pH 4.5 and 5 show a very different band pattern in SDS-PAGE. At higher molecular weights (>75 kDa), no bands were visible, as was the case for the supernatant. Instead, an intense staining is seen for proteins with a lower molecular weight (10 kDa). This staining can also be seen clearly for the proteins in the supernatant. It cannot be said whether the peptides with a low molecular weight were proteins from another protein fraction that could have precipitated in higher amounts at higher pH values, or whether they were degradation products that occurred when proteins were broken down by the cell’s own enzymes. In the literature, it is described that some proteases are activated at pH 5. In order to prevent possible degradation by proteases and to make the samples as comparable as possible, measures had already been taken: All protein samples had been resuspended at pH 7 after precipitation before they were dried, since no enzyme activity is expected at this point [21]. Nevertheless, larger quantities of small peptides precipitated at pH 4.5 and 5.0.

The results of the SDS-PAGE indicate that the different precipitation pH values led to changes in the protein molecules. But the possibility that different fractions were precipitated at different pH values cannot be completely ruled out. Therefore, both explanations must continue to be kept in mind when investigating the influence of precipitation pH on protein properties.

### 3.3. Influence of the Precipitation pH on Solubility and Aggregation Behavior of the Proteins

**Figure 3:**
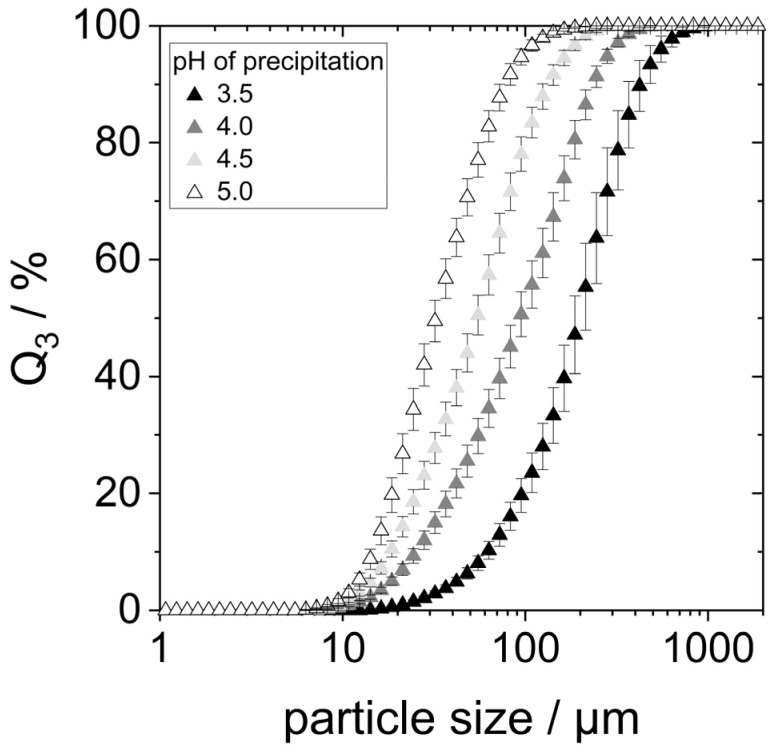
Particle size distribution of protein aggregates after 2 h stirring in water at pH 7.

Acid denaturation will cause the unfolding of the protein structure, exposing hydrophobic groups previously buried within the molecule. This can result in various effects, one of them being the agglomeration of proteins due to hydrophobic interactions. To check whether this was the case for the prepared samples, especially those precipitated at pH 3.5 and 4, the particle size of lyophilized yeast proteins redispersed in water at pH 7 for two hours on the shaking table was determined using static light scattering (SLS). The particle size distributions are shown in Figure 3.

SLS detected particles between 10 µm to approximately 500 µm in all four samples, indicating the presence of protein agglomerates. The particle size distributions showed smaller agglomerates with increasing precipitation pH within the considered pH range. The d_90,3_-value of the proteins precipitated at pH 3.5 is around 450 µm, while the d_90,3_-value for the precipitation pH of 5.0 is only around 75 µm.

Hydrophobic molecules are generally less soluble in polar media like water. Therefore, another way to evaluate the impact of precipitation pH on the molecule’s hydrophobicity, and thus to verify acid denaturation, is the measurement of protein solubility. For this purpose, the protein concentration of the redispersed lyophilized proteins was determined after 2 h of dissolution in water at pH 7 and subsequent centrifugation of the insoluble protein aggregates using the Bradford method and related to the weighed amount of protein.

**Figure 4:**
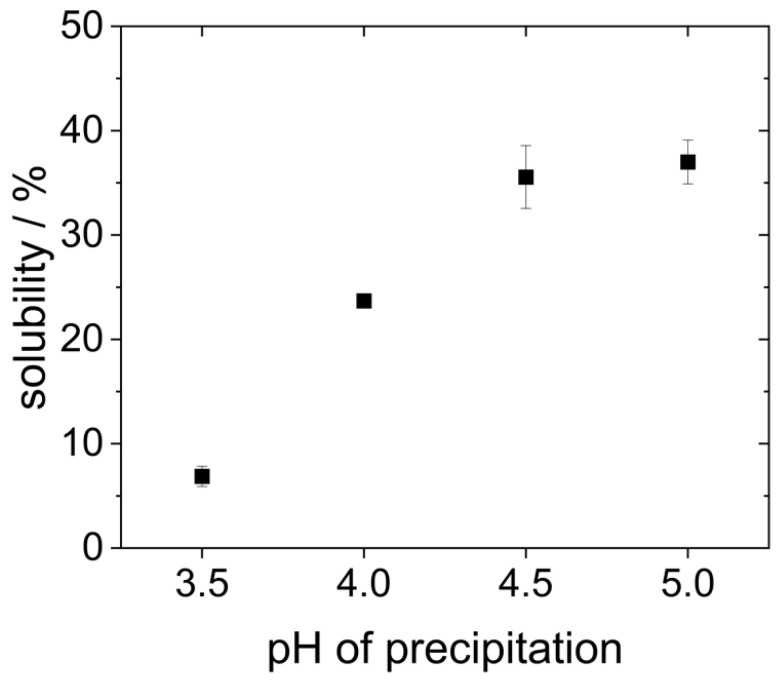
Solubility of proteins after 2 h in water at pH 7 on the shaking table after precipitation at different pH values.

As can be seen in Figure 4, the proteins that were precipitated at pH 4.5 and 5.0 showed the highest solubility (around 35%). However, it is already known from the literature that yeast proteins are poorly soluble in water due to their high hydrophobicity [19,20,27]. Reduced precipitation pH resulted in an even lower protein solubility. The solubility of the proteins that were precipitated at pH 4.0 only showed a solubility of 25%. The proteins that were precipitated at pH 3.5 were the least soluble. Here, the solubility was only around 7%.

The solubility at pH 7 presented in Figure 4 differs significantly from previous studies. For example, Pacheco and Sgarbieri describe a solubility of 50% at pH 7 for yeast proteins precipitated at pH 4.2, whereas Lee et al. reported a solubility of only 20% for proteins precipitated at pH 4.4. These differences can result from the fact that different methods were used to determine the protein content, e.g., Pacheco and Sgarbieri used the Kjeldahl method instead of the Bradford assay employed by Lee et al., and in the present study. Moreover, different types or strains of yeast might be characterized by different inherent protein solubility. In the study of Lee et al., dry yeast was used instead of fresh yeast [18,30]. 

Both the larger agglomerates (s. Figure 3) and the reduced solubility (s. Figure 4) indicate that the proteins precipitated at pH 3.5 and 4.0 have a higher hydrophobicity than those precipitated at higher pH. This might indeed be the result of hydrophobic regions having been exposed by acid denaturation and resulting unfolding of the molecular structure [8,20]. Moreover, it can clearly be seen that even when lyophilized yeast proteins are dissolved at neutral pH, the precipitation pH still has an influence on their colloidal properties. Consequently, effects on the proteins’ technofunctional properties are also expected. This is going to be explored in the following chapters.

### 3.4. Influence of the Precipitation pH on the Emulsion-Stabilizing Properties

All 0.95 wt% yeast protein solutions were able to stabilize emulsions with 5 wt% rapeseed oil. On the day of preparation, all emulsions had a creamy white color and visually appeared homogeneous (see Figure S2, Supplementary Materials).

**Figure 5:**
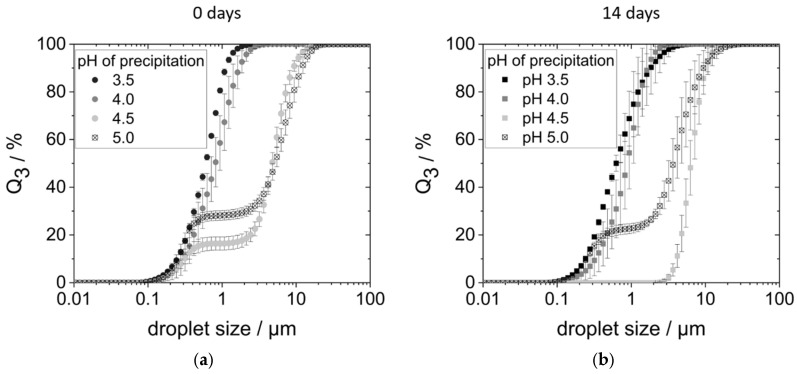
Droplet size distribution of the emulsions: (**a**) on the day of preparation; (**b**) after 14 days of storage in the refrigerator. The emulsions were prepared with 0.95 wt% protein, precipitated at pH 3.5–5.0, and redissolved in water at pH 7 and 5 wt% rapeseed oil in a high-pressure homogenizer at 400 bar.

Figure 5a shows the droplet size distributions (DSDs) of the corresponding emulsions on the day of production. Emulsions produced with proteins precipitated at pH 3.5 and 4.0 had monomodal distributions with droplet sizes between 0.1 and 3 µm. On the other hand, the DSDs of the emulsions with proteins precipitated at pH 4.5 and 5.0 were bimodal. In each case, a smaller proportion of the droplets ranged between 0.1 and 0.5 µm, while oil droplets of the second fraction had sizes between 2 and 20 µm.

The larger droplet sizes measured, i.e., the second fraction in the size range of 2 to 20 µm, could either be larger droplets due to coalescence. However, they could also be flocks of agglomerated but still individually stabilized droplets. To check this, the emulsions were examined under the microscope. Indeed, both larger droplets (around 5 µm) as well as droplet agglomerates (around 20 µm) consisting of smaller individual droplets could be observed.

At a precipitation pH of 3.5, however, only small, individual droplets were observed. Figure S3 in the Supplementary Materials shows a comparison of the microscopic images of emulsions stabilized with proteins precipitated at pH 3.5 and 4.5. The visual aspect of pH 4 emulsions was comparable to the pH 3.5 sample. Just as much, emulsions stabilized with proteins precipitated at pH 4.5 and 5.0 gave the same visual impression.

In none of the emulsions (Figure 5), particles larger than 20 µm—that is, the size of the measured protein aggregates (see Figure 3)—were found. This shows that proteins did not further agglomerate during emulsification. Instead, it points towards the fact that protein aggregates are redispersed during the emulsification process. The hydrophobic oil interface present during emulsification most likely allows the protein molecules to adsorb immediately, preventing strong reaggregation.

After 14 days of storage in the refrigerator, the emulsions prepared with proteins precipitated at pH 4.0, 4.5, and 5.0 creamed (see Figure S2 (right) in the Supplementary Materials), with creaming being most pronounced in the pH 4.5 sample. Emulsions prepared with proteins precipitated at pH 3.5 did not show any creaming.

Over the course of 2 weeks, there was hardly a change in the DSDs of emulsions prepared with proteins precipitated at pH 3.5, pH 4.0, and pH 5.0 (see Figure 5b). Only the DSD of the pH 4.5 sample changed: The small droplet fraction could no longer be detected. Instead, a monomodal distribution with large droplets between 2 and 20 µm was measured.

In summary, yeast proteins precipitated at pH 4.5 and 5.0 were characterized by smaller detected molecular weights, smaller aggregate sizes, and better solubility. Yet, in comparison to the other two protein samples (precipitation pH 3.5 and 4.0), they were only able to stabilize larger oil droplets that moreover agglomerated. The reason for this might be insufficient steric stabilization of the oil droplets covered by yeast proteins precipitated at pH 4.5 and 5.0. Smaller molecules generally show faster adsorption kinetics and are able to quickly adsorb at the oil–water interface. However, when there is a lack of electrostatic stabilization, small emulsifying molecules might not provide enough steric stabilization to the interface. This might result in droplet coalescence or aggregation which was indeed observed here. Later, the larger droplets or droplet agglomerates caused faster creaming of the emulsions, so that the samples were not stable during the 14 days of storage (Figure S2 in the Supplementary Materials).

In contrast, yeast proteins precipitated at pH 3.5 and 4.0 were characterized by larger detected molecular weights, larger aggregate sizes, and worse solubility. Yet, they were able to stabilize smaller and individual oil droplets. This highlights acid denaturation, causing exposition of hydrophobic groups and molecule aggregation. Proteins with higher surface hydrophobicity possess a higher interfacial affinity, resulting in a faster and more efficient stabilization of oil droplets. However, given the fact that steric stabilization of the oil droplets is most likely of relevance, size effects come into play as well. On the one hand, the lower pH values of 3.5 and 4.0 might have caused the precipitation of larger protein fractions that are then able to better stabilize the oil droplet interface sterically. To further analyze this, a more detailed investigation of the individual protein fractions is necessary and will be conducted in the future. On the other hand, the molecules precipitated at pH 3.5 and 4.0 might have had the same initial size as those precipitated at pH 4.5 and 5.0. However, due to acid denaturation, they might have aggregated so strongly that the resulting aggregates were perceived as one large molecule. This assumption is not contradicted by the SDS-PAGE results showing larger molecular sizes for the pH 3.5 and 4.0 samples because non-reducing conditions were chosen for the SDS-PAGE. As a result, aggregation caused by disulfide bonding would not be detected as such.

However, protein aggregates can be broken up under harsh homogenization conditions. When high shear forces are applied to protein aggregates, they can be separated into their individual molecules and might stay separated or rearrange into aggregates of different sizes. This will have effects on all investigated parameters, i.e., detectable solubility, aggregate size, and emulsifying properties. Whether the precipitation at pH 3.5 and 4.0 resulted in precipitation of larger molecules or predominantly in the formation of larger aggregates will thus be investigated by applying an additional homogenization step to the continuous phase. The results are presented in the next chapter.

### 3.5. Influence of an Additional Homogenization Step Before Emulsification on Protein Solubility and Agglomeration

**Figure 6:**
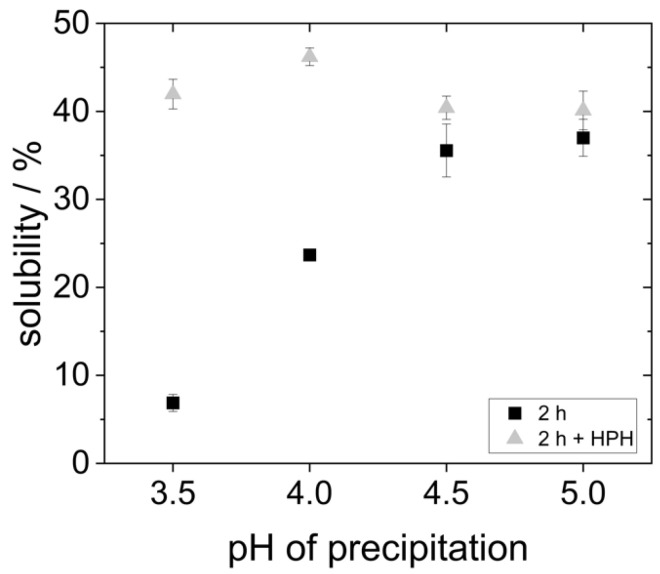
Solubility of proteins precipitated at different pH values and, after lyophilization, dissolved in water at pH 7 for 2 h, and partly with subsequent HPH treatment at 400 bar.

In order to investigate the extent of protein aggregate formation, an additional treatment of the continuous phase was carried out with the high-pressure homogenizer at 400 bar before emulsification. Figure 6 shows the solubility of the proteins with subsequent high-pressure homogenization (HPH)-treatment of the continuous phase in comparison to the data obtained for proteins without the HPH treatment.

The results show that after HPH treatment, the solubility of all yeast protein samples is 40–45%. This represents a significant improvement in the solubility of the proteins that were precipitated at more acidic pH values. However, for the proteins that were precipitated at pH 5.0, the solubility could not be increased any further by HPH treatment. The solubility data suggest that agglomerates in the samples at pH 3.5 and pH 4.0 were comminuted to smaller sizes, resulting in a better overall solubility.

**Figure 7:**
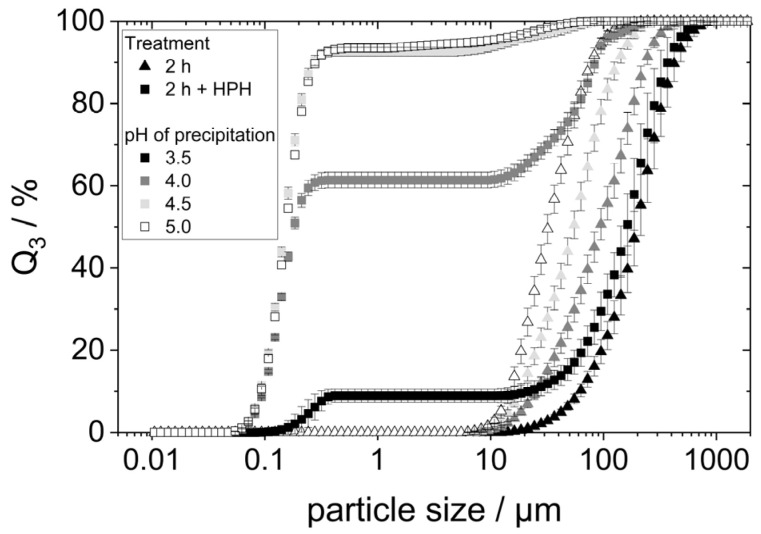
Particle size distribution of the protein aggregates after HPH treatment (400 bar) and, for comparison, the particle sizes after only 2 h in water at pH 7 on the shaking table.

To verify that the additional dispersion step indeed comminuted agglomerates, the protein agglomerate size was measured using SLS. Figure 7 compares the particle size distributions to those measured in the samples without an additional homogenization step.

The HPH treatment led to different results for the different precipitated proteins. For the proteins precipitated at pH 3.5, the PSD changed only minimally. A small fraction of the particles possessed a very small size between 0.1 and 0.4 µm. The remaining particles maintained a size between 10 and 1000 µm. This indicates that only a small portion of the agglomerates was comminuted in the additional HPH-treatment. The aggregates of the proteins precipitated at higher pH values were much more comminuted: samples with pH 4.5 and pH 5.0 exhibited almost entirely very small particle sizes below 0.5 µm. The comminution of the protein agglomerates of the pH 4.0 sample seemed to have been comminuted slightly less effectively. Nevertheless, the majority of particles in that sample possessed sizes smaller than 0.5 µm.

The results show that high-pressure homogenization of the lyophilized, redispersed proteins can breakup occurring agglomerates and can thus lead to a finer dispersion of protein agglomerates. However, the results also indicate that highly hydrophobic proteins reassemble into larger aggregates after having been broken up in the high-pressure homogenizer. This can be concluded from the fact that the samples precipitated at pH 3.5 and pH 4.0 (those having experienced acid denaturation) show a distinct fraction of very small particles as well as a fraction of larger particles close to their initial size. In the case of an insufficient comminution of agglomerates, one would expect a much broader particle size distribution and particles in the medium size range > 0.5 µm, instead.

### 3.6. Influence of an Additional Homogenization Step Before Emulsification on Emulsion Stabilization

**Figure 8:**
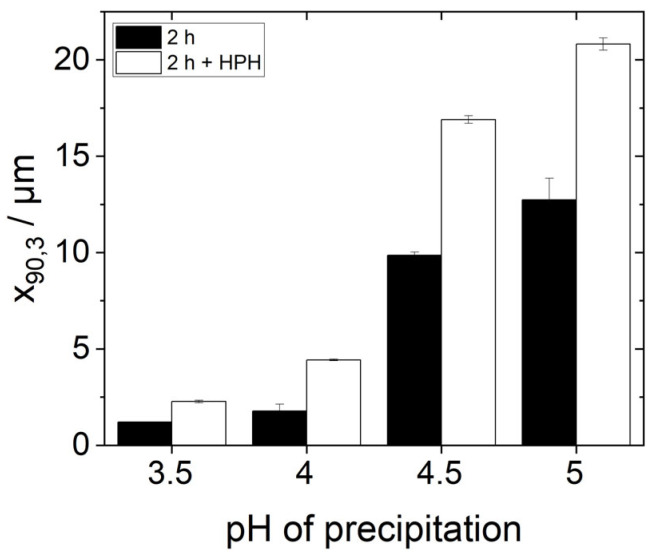
The 90th percentile diameter d_90,3_ values of the droplet size distributions of emulsions on the day of preparation, produced with 0.95 wt% protein (precipitated at pH 3.5–5.0 and redissolved in water at pH 7, partially with subsequent high-pressure homogenization (HPH)-treatment) and 5 wt% rapeseed oil in a high-pressure homogenizer at 400 bar.

In order to investigate the influence of the additional homogenization step and thus of finer dispersed protein agglomerates on emulsification, emulsions were produced and characterized as previously described using the high-pressure homogenized protein dispersion as continuous phase. Figure 8 shows the d_90,3_-values of these emulsions in comparison to the d_90,3_-values of the emulsions without an additional HPH-step. It can be seen that the characteristic droplet sizes d_90,3_ of all the emulsions with an additional homogenization step are larger than those without the additional treatment. Thus, additional HPH-treatment did not improve but instead worsened the emulsion-stabilizing properties of the proteins.

In order to understand this phenomenon, some points need to be kept in mind. First, the solubility of all protein samples was more or less equal after HPH-treatment (Figure 6). Yet, the stabilized oil droplet sizes differed (Figure 8). Consequently, protein solubility as measured by the Bradford assay is not a reliable method to predict the emulsifying properties of yeast proteins after isoelectric precipitation. Secondly, the protein particle size measured by SLS in water does not alone predict the emulsifying properties of yeast proteins either. The order of the particle size (s. Figure 3) correlates inversely with the d_90,3_-values in the emulsions in Figure 8. However, this is not the case for the samples with additional HPH-treatment, as can be seen when comparing Figure 7 and Figure 8. The most likely reason for this observation is that the real particle size of the proteins involved in emulsion stabilization could not, or at least not in all cases be detected by SLS due to the reassembly into larger agglomerates in water as a hydrophilic solvent.

Even if the protein particles reassembled into larger aggregates after breaking up in the high-pressure homogenizer before measurement, some of them remained broken down. The highly crushed particles, in turn, could make the interfacial film around the droplets during emulsification less stable. This could explain the poorer stabilization of small oil drops, as can be seen by the increase in the d_90,3_-values in Figure 8. This is supported by the fact that a significant break-up of the agglomerates (Figure 7) and at the same time a significant increase in the respective d_90,3_ values was measured, especially for the precipitation pH values 4.5 and 5.0.

Another explanation for the deterioration in stabilization after HPH-treatment could be that the rearrangement of the broken aggregates in the aqueous environment to larger aggregates led to a solidification of the particles. Stronger hydrophobic interactions between the protein molecules after a rearrangement could have resulted in the hydrophobic molecular regions being exposed less when subjected to renewed stress in the high-pressure homogenizer during the emulsification process, and thus no longer being available to the same extent for attachment to the oil interface. Regardless of which mechanism can explain the poorer stabilization of small oil droplets, it is obviously not beneficial to subject the continuous phase to an HPH treatment before the actual emulsification process.

## 4. Conclusions

This study investigated the influence of the precipitation pH during the isolation of intracellular yeast proteins on the solubility, aggregation, and, above all, emulsion-stabilizing properties of these proteins. For this, four different pH values around the IEP of the yeast proteins were chosen for precipitation. In contrast to previous studies, not only pH values directly at or slightly above the IEP (pH 4.5 and 5.0) were investigated, but also slightly lower pH values (pH 3.5 and 4.0). It was shown that proteins precipitated at pH 3.5 and 4 could stabilize particularly small oil droplets and that the emulsions remained stable even after storage for 14 days. It was concluded that at these precipitation pH values, acid denaturation occurred, leading to unfolding of the proteins and to the exposure of hydrophobic and reactive molecular regions from the interior of the globular molecule. This resulted in higher molecular weights of the precipitated proteins, lower solubility, and more agglomeration, but also much improved emulsifying properties. However, it cannot entirely be ruled out that the observed differences are the result of different protein fractions with different solubility or interface affinity having precipitated at lower pH values. Nevertheless, it was shown that it is worthwhile to deviate from the precipitation at pH values greater than 4.0 currently common in the literature in order to obtain proteins with improved emulsion-stabilizing properties.

## Data Availability

The original contributions presented in this study are included in the article/Supplementary Materials. Further inquiries can be directed to the corresponding author.

## Supplementary Materials

The following supporting information can be downloaded at: https://www.mdpi.com/article/10.3390/foods14152643/s1, Figure S1. Zeta potential of the supernatant after cell disruption and centrifugation at different pH values; Figure S2. Emulsions (a) on the day of production and (b) after 14 days; Figure S3. Microscope images of the emulsions on the day of preparation.

## Abbreviations

The following abbreviations are used in this manuscript:

IEP	Isoelectric point
HPH	High-pressure homogenizer
PSD	Particle size distribution
DSD	Droplet size distribution
ANOVA	Analysis of variance
SDS-PAGE	Sodium dodecyl sulfate polyacrylamide gel electrophoresis
