# Supplementary material for: pH-Controlled Yeast Protein Precipitation from Saccharomyces cerevisiae: Acid-Induced Denaturation for Improved Emulsion Stability
Source: Foods. 2025 Jul 28;14(15):2643. doi: 10.3390/foods14152643 (PMC12346520; doi:10.3390/foods14152643)
Supplement: Supplementary file 1 [file foods-14-02643-s001.zip › foods-3782626-supplementary.pdf]

Supplementary Materials

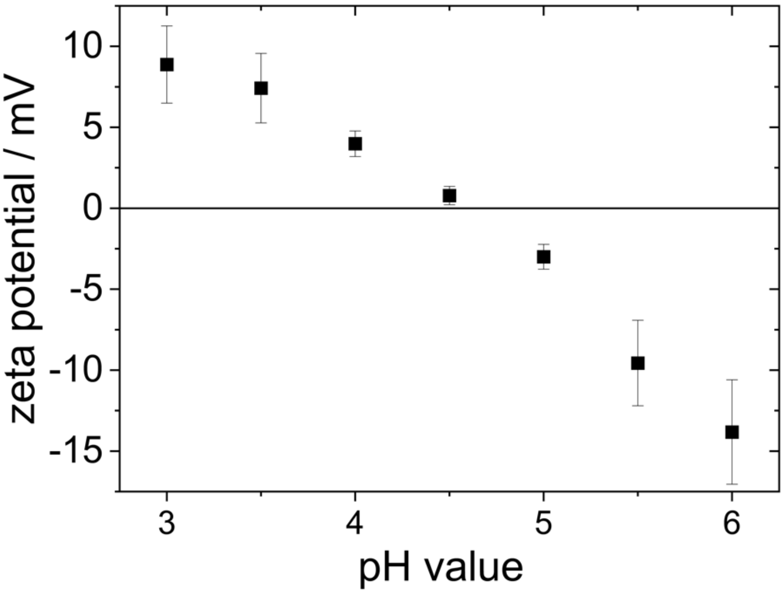

Figure S1. Zeta potential of the supernatant after cell disruption and centrifugation at different pH values.

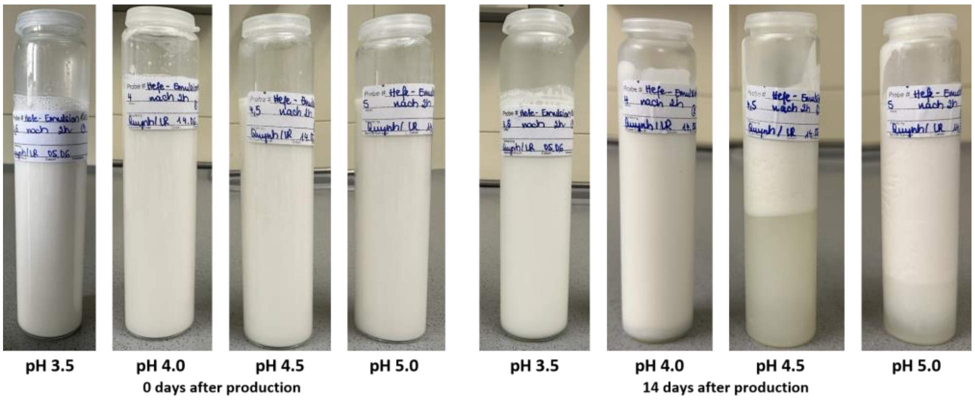

(**a**) (**b**)

Figure S2. Emulsions **(a)** on the day of production and **(b)** after 14 days. Dissolved at pH 7 for 2 hours and then emulsified in HPH at 400 bar. 0.95 wt% protein and 5 wt% oil.

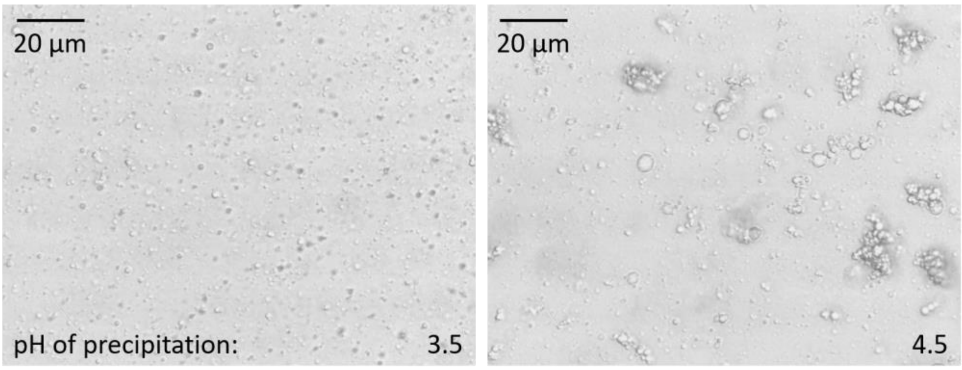

(**a**) (**b**)

Figure S3. Microscope images of the emulsions on the day of preparation, prepared at 400 bar in a high-pressure homogenizer with 5% oil and 0.95% protein precipitated **(a)** at pH 3.5 and **(b)** at 4.5.
